# Supplementary material for: Evaluation of Deep Learning Model Architectures for Point-of-Care Ultrasound Diagnostics
Source: Bioengineering (Basel). 2024 Apr 18;11(4):392. doi: 10.3390/bioengineering11040392 (PMC11048259; doi:10.3390/bioengineering11040392)
Supplement: Supplementary file 1 [file bioengineering-11-00392-s001.zip › bioengineering-2952300-supplementary.pdf]

Supplementary Information

Table S1. Summary of top 3 performing architecture hyperparameter configurations based on Bayesian optimization for each scan site.

		CNN Depth	Node Size	Multiplier	Filter Size	Dropout	Accuracy
RUQ	1st	4	13	1.99356	7	3	0.867
	2nd	5	16	1.96767	7	8	0.859
	3rd	5	16	1.93826	7	8	0.857
BLD	1st	2	2	1.61756	6	2	0.575
	2nd	4	10	1.91307	7	5	0.57
	3rd	5	11	1.86532	2	8	0.569
PTX_B	1st	3	16	1.82879	5	1	0.747
	2nd	3	13	1.02141	2	5	0.744
	3rd	3	16	1.17496	4	1	0.74
PTX_M	1st	2	16	1.00818	3	3	0.86
	2nd	2	2	1.47797	3	9	0.853
	3rd	2	12	1.02064	3	3	0.845
HTX_B	1st	4	16	1.008018	3	6	0.854
	2nd	4	13	1.941593	5	6	0.842
	3rd	5	16	1.677502	2	3	0.836
HTX_M	1st	3	16	1.832214	2	9	0.874
	2nd	4	16	1.884837	2	9	0.866
	3rd	3	16	1.192284	2	9	0.844

Table S2. Blind test performance metric summary for each model architecture for the RUQ scan site. Results are shown for each metric as mean values across the 5 LOSO runs with standard deviation shown in parentheses. Heat map overlay is setup for green coloring to indicate the stronger performing model for each row, metric.

RUQ Scan Site Results							
	Simple	Optimized 1	Optimized 2	Optimized 3	ShrapML	MobileNetV2	DarkNet53
Accuracy	0.71(±0.07)	0.7(±0.08)	0.66(±0.09)	0.72(±0.09)	0.73(±0.08)	0.74(±0.09)	0.67(±0.13)
AUROC	0.78(±0.12)	0.76(±0.05)	0.74(±0.06)	0.76(±0.11)	0.81(±0.06)	0.79(±0.06)	0.72(±0.16)
F1	0.71(±0.08)	0.72(±0.06)	0.68(±0.09)	0.72(±0.09)	0.75(±0.06)	0.73(±0.14)	0.65(±0.22)
Precision	0.72(±0.15)	0.73(±0.18)	0.68(±0.18)	0.74(±0.16)	0.75(±0.16)	0.77(±0.14)	0.67(±0.19)
Recall	0.74(±0.2)	0.76(±0.19)	0.74(±0.2)	0.74(±0.16)	0.79(±0.14)	0.72(±0.22)	0.7(±0.31)
Specificity	0.67(±0.2)	0.64(±0.3)	0.58(±0.27)	0.69(±0.24)	0.68(±0.26)	0.76(±0.18)	0.64(±0.26)

*Results shown as Average (± Standard Deviation)

Table S3. Blind test performance metric summary for each model architecture for the BLD scan site. Results are shown for each metric as mean values across the 5 LOSO runs with standard deviation shown in parentheses. Heat map overlay is setup for green coloring to indicate the stronger performing model for each row, metric.

BLD Scan Site Results							
	Simple	Optimized 1	Optimized 2	Optimized 3	ShrapML	MobileNetV2	DarkNet53
Accuracy	0.52(±0.09)	0.57(±0.10)	0.58(±0.11)	0.57(±0.10)	0.62(±0.14)	0.60(±0.12)	0.57(±0.17)
AUROC	0.52(±0.15)	0.57(±0.13)	0.54(±0.13)	0.56(±0.12)	0.64(±0.20)	0.59(±0.21)	0.56(±0.24)
F1	0.45(±0.15)	0.58(±0.13)	0.60(±0.16)	0.60(±0.13)	0.56(±0.21)	0.57(±0.20)	0.55(±0.21)
Precision	0.52(±0.16)	0.57(±0.08)	0.57(±0.10)	0.56(±0.09)	0.62(±0.16)	0.59(±0.15)	0.58(±0.19)
Recall	0.41(±0.15)	0.63(±0.22)	0.67(±0.29)	0.67(±0.24)	0.54(±0.26)	0.59(±0.27)	0.55(±0.24)
Specificity	0.63(±0.14)	0.52(±0.22)	0.5(±0.23)	0.47(±0.21)	0.70(±0.14)	0.62(±0.19)	0.59(±0.26)
*Results shown as Average (± Standard Deviation)							

Table S4. Blind test performance metric summary for each model architecture for the PTX_B scan site. Results are shown for each metric as mean values across the 5 LOSO runs with standard deviation shown in parentheses. Heat map overlay is setup for green coloring to indicate the stronger performing model for each row, metric.

PTX_B Scan Site Results							
	Simple	Optimized 1	Optimized 2	Optimized 3	ShrapML	MobileNetV2	DarkNet53
Accuracy	0.67(±0.10)	0.49(±0.01)	0.68(±0.12)	0.58(±0.11)	0.65(±0.12)	0.68(±0.12)	0.60(±0.12)
AUROC	0.79(±0.11)	0.49(±0.01)	0.79(±0.14)	0.62(±0.13)	0.68(±0.19)	0.73(±0.16)	0.66(±0.15)
F1	0.73(±0.06)	0.60(±0.12)	0.74(±0.09)	0.64(±0.06)	0.6(±0.15)	0.72(±0.07)	0.55(±0.27)
Precision	0.65(±0.12)	0.49(±0.02)	0.64(±0.12)	0.61(±0.15)	0.69(±0.17)	0.67(±0.14)	0.60(±0.13)
Recall	0.86(±0.10)	0.49(±0.50)	0.88(±0.08)	0.77(±0.24)	0.55(±0.20)	0.79(±0.11)	0.60(±0.35)
Specificity	0.49(±0.26)	0.49(±0.50)	0.49(±0.22)	0.38(±0.42)	0.74(±0.15)	0.56(±0.30)	0.6(±0.35)
*Results shown as Average (± Standard Deviation)							

Table S5. Blind test performance metric summary for each model architecture for the PTX_M scan site. Results are shown for each metric as mean values across the 5 LOSO runs with standard deviation shown in parentheses. Heat map overlay is setup for green coloring to indicate the stronger performing model for each row, metric.

PTX_M Scan Site Results							
	Simple	Optimized 1	Optimized 2	Optimized 3	ShrapML	MobileNetV2	DarkNet53
Accuracy	0.66(±0.07)	0.73(±0.10)	0.62(±0.14)	0.73(±0.13)	0.64(±0.15)	0.89(±0.04)	0.91(±0.05)
AUROC	0.73(±0.11)	0.82(±0.13)	0.65(±0.15)	0.80(±0.16)	0.71(±0.17)	0.97(±0.02)	0.96(±0.04)
F1	0.68(±0.09)	0.75(±0.10)	0.60(±0.19)	0.74(±0.13)	0.65(±0.15)	0.89(±0.04)	0.91(±0.05)
Precision	0.65(±0.06)	0.69(±0.07)	0.61(±0.13)	0.70(±0.11)	0.66(±0.18)	0.91(±0.08)	0.88(±0.09)
Recall	0.73(±0.18)	0.82(±0.14)	0.61(±0.24)	0.79(±0.16)	0.67(±0.20)	0.88(±0.06)	0.96(±0.03)
Specificity	0.60(±0.14)	0.63(±0.07)	0.63(±0.12)	0.66(±0.13)	0.62(±0.23)	0.90(±0.09)	0.86(±0.11)
*Results shown as Average (± Standard Deviation)							

Table S6. Blind test performance metric summary for each model architecture for the HTX_B scan site. Results are shown for each metric as mean values across the 5 LOSO runs with standard deviation shown in parentheses. Heat map overlay is setup for green coloring to indicate the stronger performing model for each row, metric.

HTX_B Scan Site Results							
	Simple	Optimized 1	Optimized 2	Optimized 3	ShrapML	MobileNetV2	DarkNet53
Accuracy	0.61(±0.07)	0.70(±0.08)	0.71(±0.07)	0.72(±0.10)	0.65(±0.13)	0.74(±0.10)	0.72(±0.10)
AUROC	0.69(±0.11)	0.78(±0.07)	0.79(±0.06)	0.82(±0.09)	0.75(±0.14)	0.83(±0.08)	0.83(±0.10)
F1	0.63(±0.08)	0.74(±0.05)	0.68(±0.14)	0.74(±0.07)	0.67(±0.10)	0.78(±0.07)	0.76(±0.06)
Precision	0.62(±0.10)	0.68(±0.11)	0.77(±0.15)	0.73(±0.15)	0.66(±0.13)	0.70(±0.13)	0.69(±0.13)
Recall	0.67(±0.16)	0.84(±0.10)	0.69(±0.25)	0.78(±0.14)	0.73(±0.18)	0.89(±0.06)	0.87(±0.13)
Specificity	0.56(±0.19)	0.56(±0.21)	0.73(±0.22)	0.66(±0.26)	0.57(±0.32)	0.58(±0.22)	0.56(±0.29)
*Results shown as Average (± Standard Deviation)							

Table S7. Blind test performance metric summary for each model architecture for the HTX_M scan site. Results are shown for each metric as mean values across the 5 LOSO runs with standard deviation shown in parentheses. Heat map overlay is setup for green coloring to indicate the stronger performing model for each row, metric.

HTX_M Scan Site Results							
	Simple	Optimized 1	Optimized 2	Optimized 3	ShrapML	MobileNetV2	DarkNet53
Accuracy	0.71(±0.11)	0.74(±0.10)	0.71(±0.13)	0.75(±0.16)	0.71(±0.10)	0.85(±0.07)	0.81(±0.08)
AUROC	0.77(±0.12)	0.81(±0.12)	0.83(±0.11)	0.82(±0.13)	0.77(±0.13)	0.92(±0.05)	0.91(±0.07)
F1	0.71(±0.11)	0.76(±0.09)	0.75(±0.10)	0.78(±0.12)	0.73(±0.08)	0.86(±0.05)	0.83(±0.07)
Precision	0.72(±0.13)	0.70(±0.07)	0.66(±0.11)	0.72(±0.14)	0.70(±0.13)	0.84(±0.13)	0.78(±0.11)
Recall	0.72(±0.17)	0.83(±0.13)	0.88(±0.11)	0.85(±0.12)	0.79(±0.10)	0.90(±0.08)	0.91(±0.13)
Specificity	0.69(±0.18)	0.64(±0.08)	0.54(±0.18)	0.65(±0.24)	0.63(±0.18)	0.80(±0.19)	0.71(±0.19)
*Results shown as Average (± Standard Deviation)							
